# Supplementary material for: Observational evidence for cloud cover enhancement over western European forests
Source: Nat Commun. 2017 Jan 11;8:14065. doi: 10.1038/ncomms14065 (PMC5260849; doi:10.1038/ncomms14065)
Supplement: Supplementary Information — Supplementary Figures. [file ncomms14065-s1.pdf]

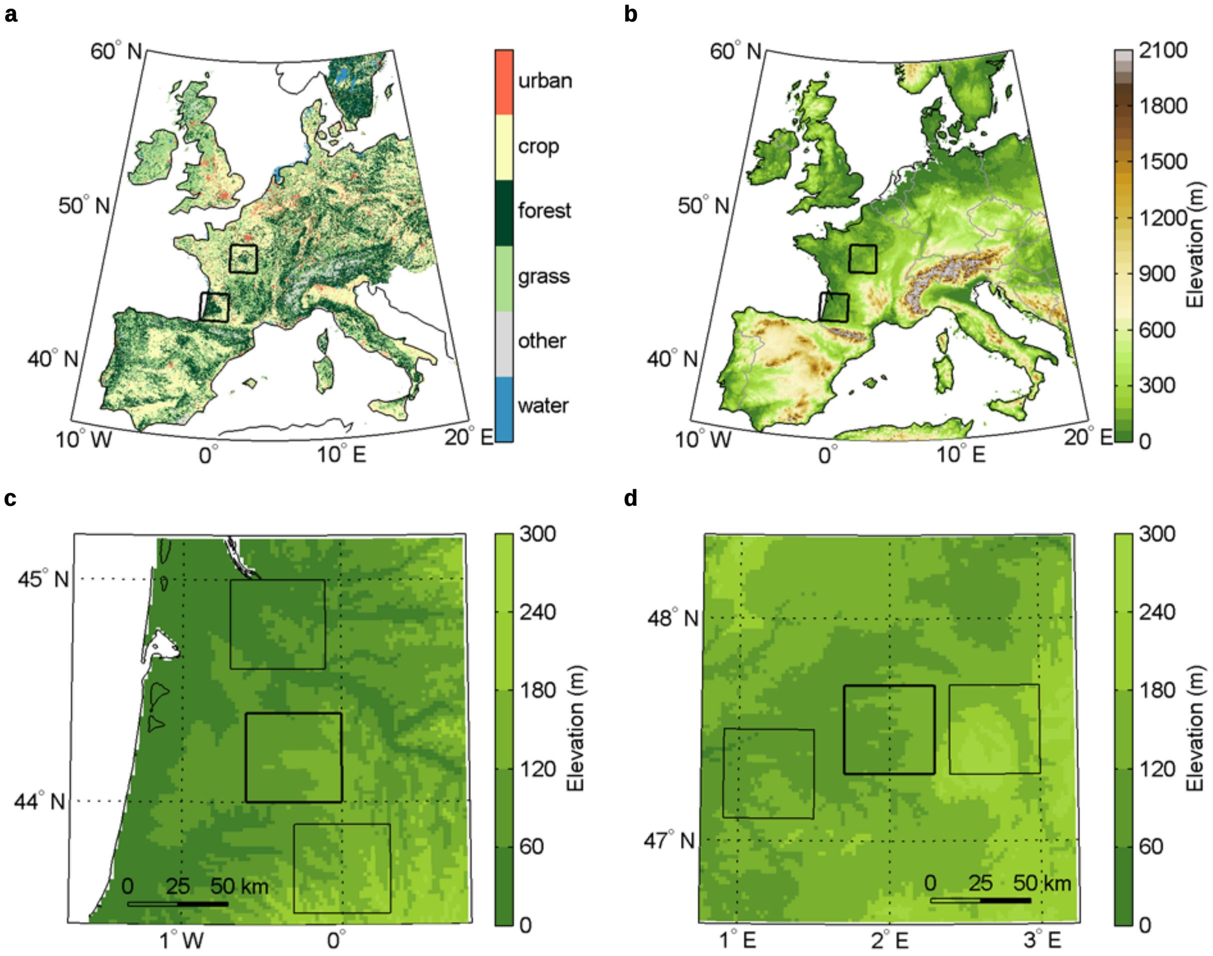

Supplementary Figure 1 | Location and characteristics of Landes and Sologne focus regions. Land use in Central-Western Europe (**a**), and elevation maps of Central-Western Europe (**b**), the Landes study region (**c**) and the Sologne study region (**d**). Note **a**–**c** use the same colour scale. The boxes in panels **a** and **b** indicate the location of the focus regions shown in **c** and **d**. The regional elevation maps show the absence of pronounced topographical features in both regions which might influence cloud formation. Boxes indicate the location of forest (thick line) and non-forest (thin line) areas for visual reference. Land use data for 2010 was taken from HILDA. Elevation data was taken from GTOPO30.

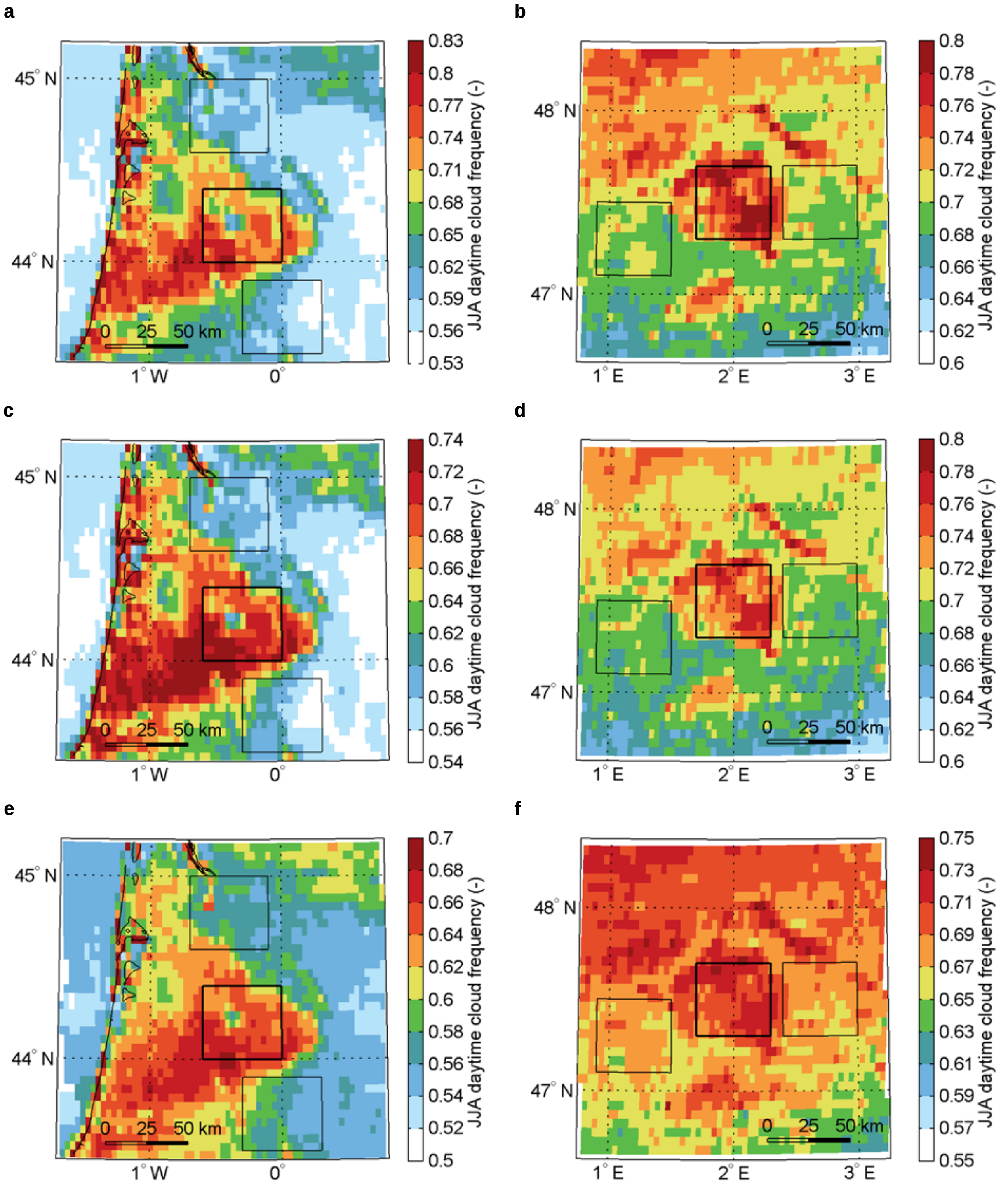

Supplementary Figure 2 | Sensitivity of summer cloud frequency to cloud optical thickness filtering in the Cloud Physical Properties algorithm. (**a,b**) June-August (JJA) cloud frequency with cloud optical thickness (COT) filter 0.15 for Landes (**a**) and Sologne (**b**). (**c,d**) JJA cloud frequency with COT filter 0.30 for Landes (**c**) and Sologne (**d**). (**e,f**) JJA cloud frequency with COT filter 0.60 for Landes (**e**) and Sologne (**f**). Shown are average cloud frequencies over the years 2004–2008. Boxes indicate the location of forest (thick line) and non-forest (thin line) areas for visual reference.

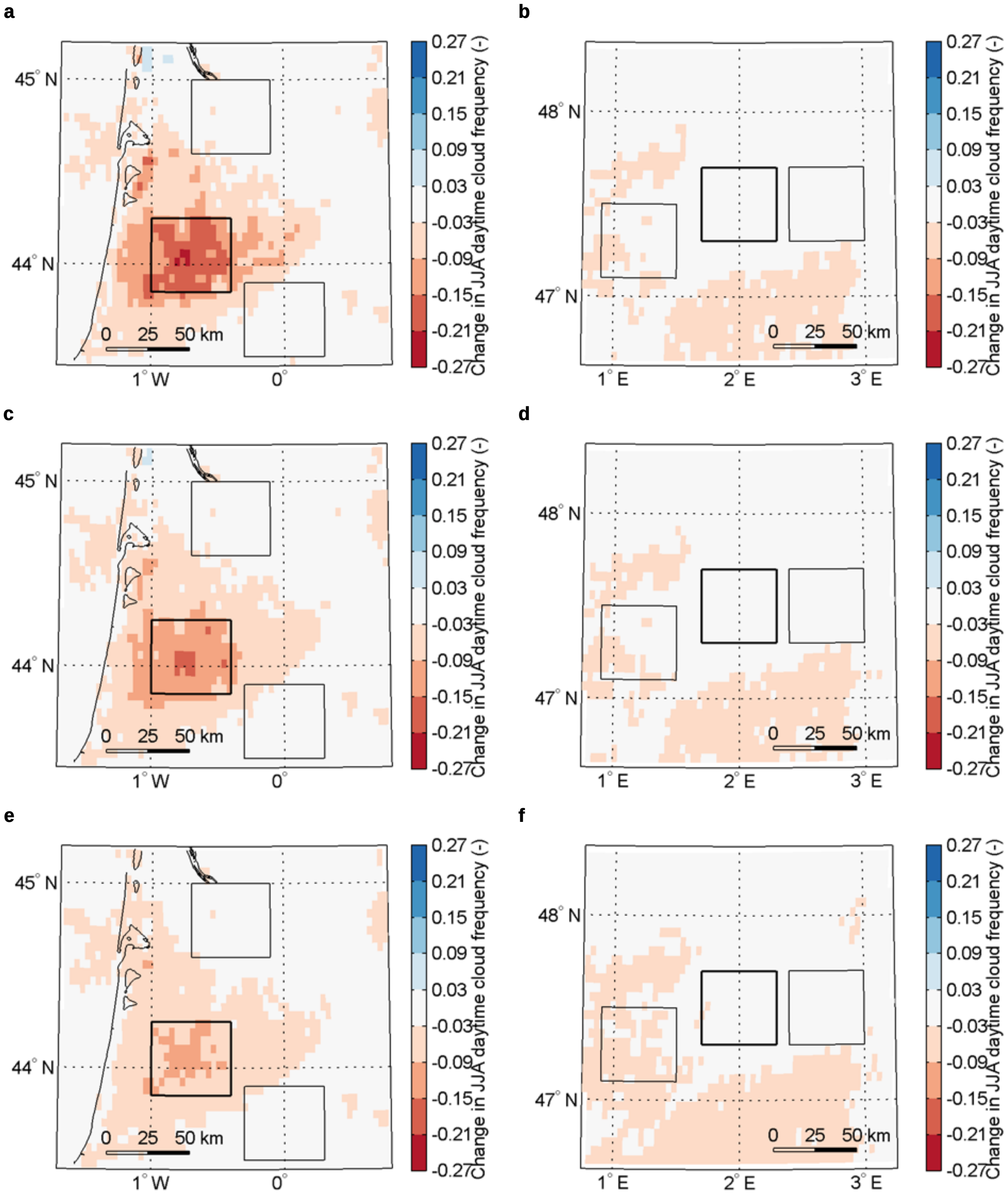

Supplementary Figure 3 | Sensitivity of difference in summer cloud frequency before and after passing of Klaus to cloud optical thickness filtering in the Cloud Physical Properties algorithm. (**a,b**) Difference in June-August (JJA) cloud frequency with cloud optical thickness (COT) filter 0.15 for Landes (**a**) and Sologne (**b**). (**c,d**) Difference in JJA cloud frequency with COT filter 0.30 for Landes (**c**) and Sologne (**d**). (**e,f**) Difference in JJA cloud frequency with COT filter 0.60 for Landes (**e**) and Sologne (**f**). Shown are differences in average cloud frequencies between the years 2004–2008 and 2009–2013. Boxes indicate the location of forest (thick line) and non-forest (thin line) areas for visual reference.

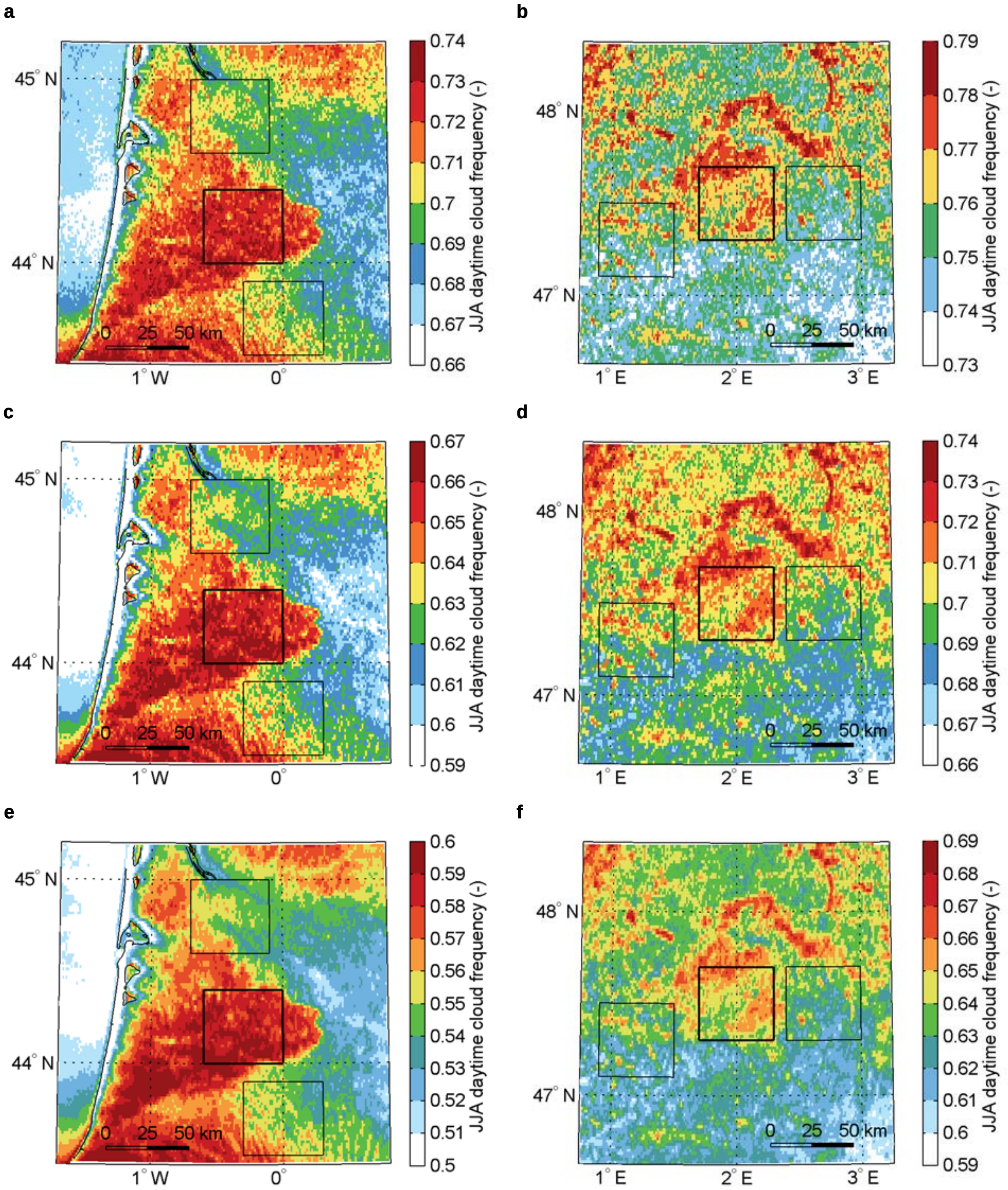

Supplementary Figure 4 | Sensitivity of summer cloud frequency to reflectance threshold in the High Resolution Visible algorithm. (**a,b**) June-August (JJA) cloud frequency with reflectance threshold of 5 for Landes (**a**) and Sologne (**b**). (**c,d**) JJA cloud frequency with reflectance threshold of 10 for Landes (**c**) and Sologne (**d**). (**e,f**) JJA cloud frequency with reflectance threshold of 20 for Landes (**e**) and Sologne (**f**). Shown are average cloud frequencies over the years 2004–2008. Boxes indicate the location of forest (thick line) and non-forest (thin line) areas for visual reference.

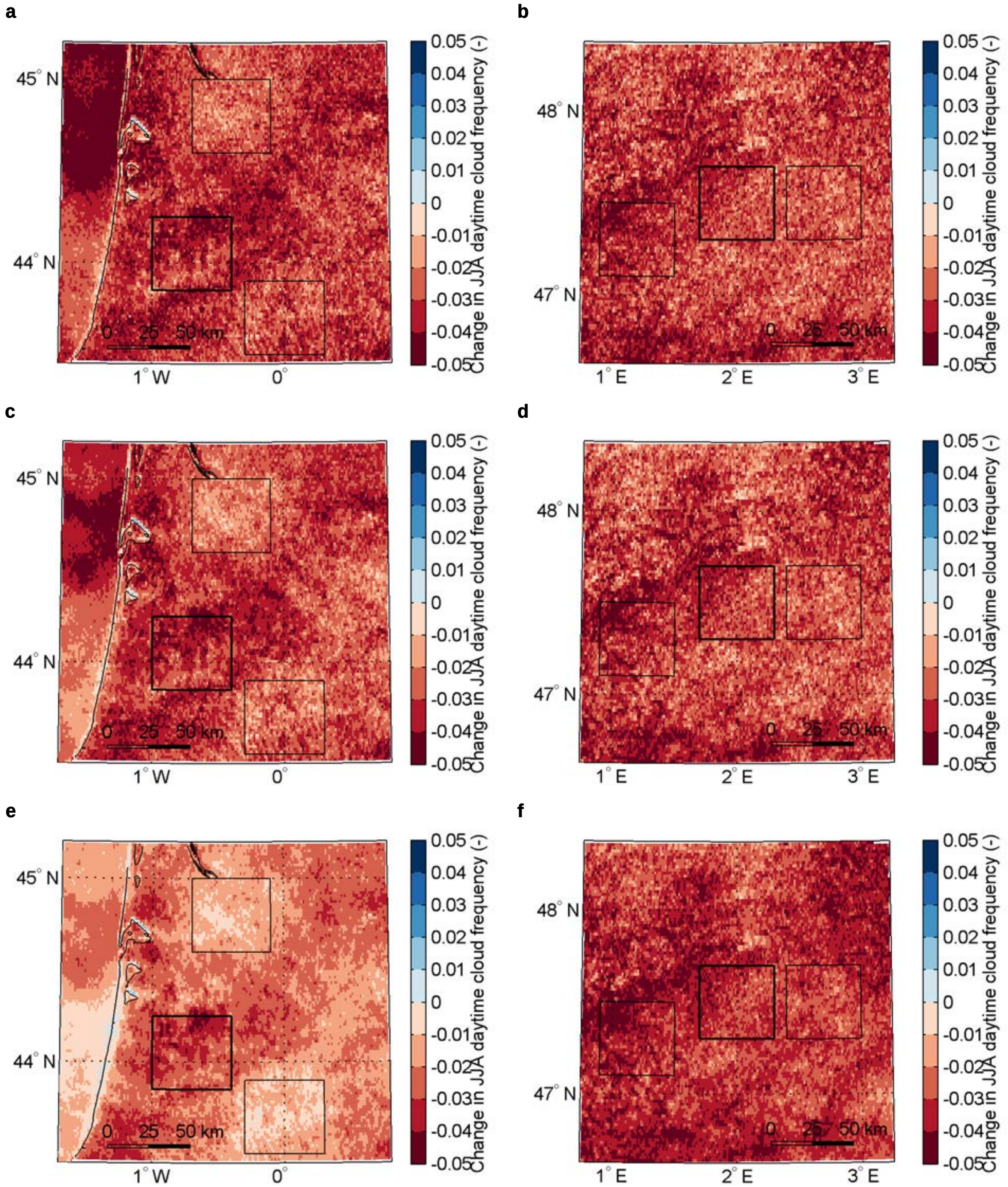

Supplementary Figure 5 | Sensitivity of difference in summer cloud frequency before and after passing of Klaus to reflectance threshold in the High Resolution Visible algorithm. (**a**,**b**) Difference in June-August (JJA) cloud frequency with reflectance threshold of 5 for Landes (**a**) and Sologne (**b**). (**c**,**d**) Difference in JJA cloud frequency with reflectance threshold of 10 for Landes (**c**) and Sologne (**d**). (**e**,**f**) Difference in JJA cloud frequency with reflectance threshold of 20 for Landes (**e**) and Sologne (**f**). Shown are differences in average cloud frequencies between the years 2004–2008 and 2009–2013. Boxes indicate the location of forest (thick line) and non-forest (thin line) areas for visual reference.

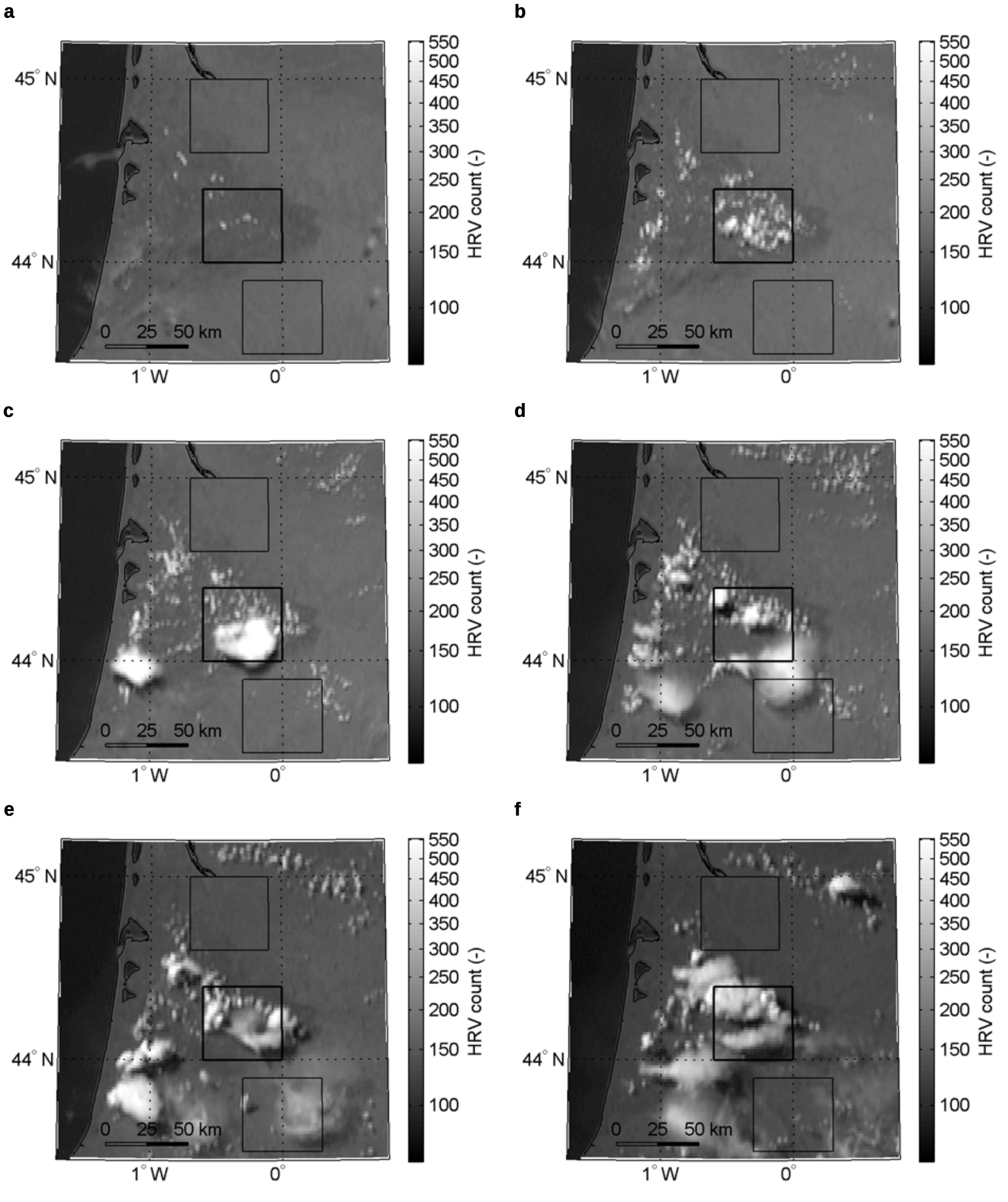

Supplementary Figure 6 | Snapshots of cloud cover development on 17 July 2006 over Landes. (**a**) 11 UTC, (**b**) 12 UTC, (**c**) 13 UTC, (**d**) 14 UTC, (**e**) 15 UTC, (**f**) 16 UTC. Images are based on Meteosat Second Generation high-resolution visible (HRV) imagery. Boxes indicate the location of forest (thick line) and non-forest (thin line) areas for visual reference.

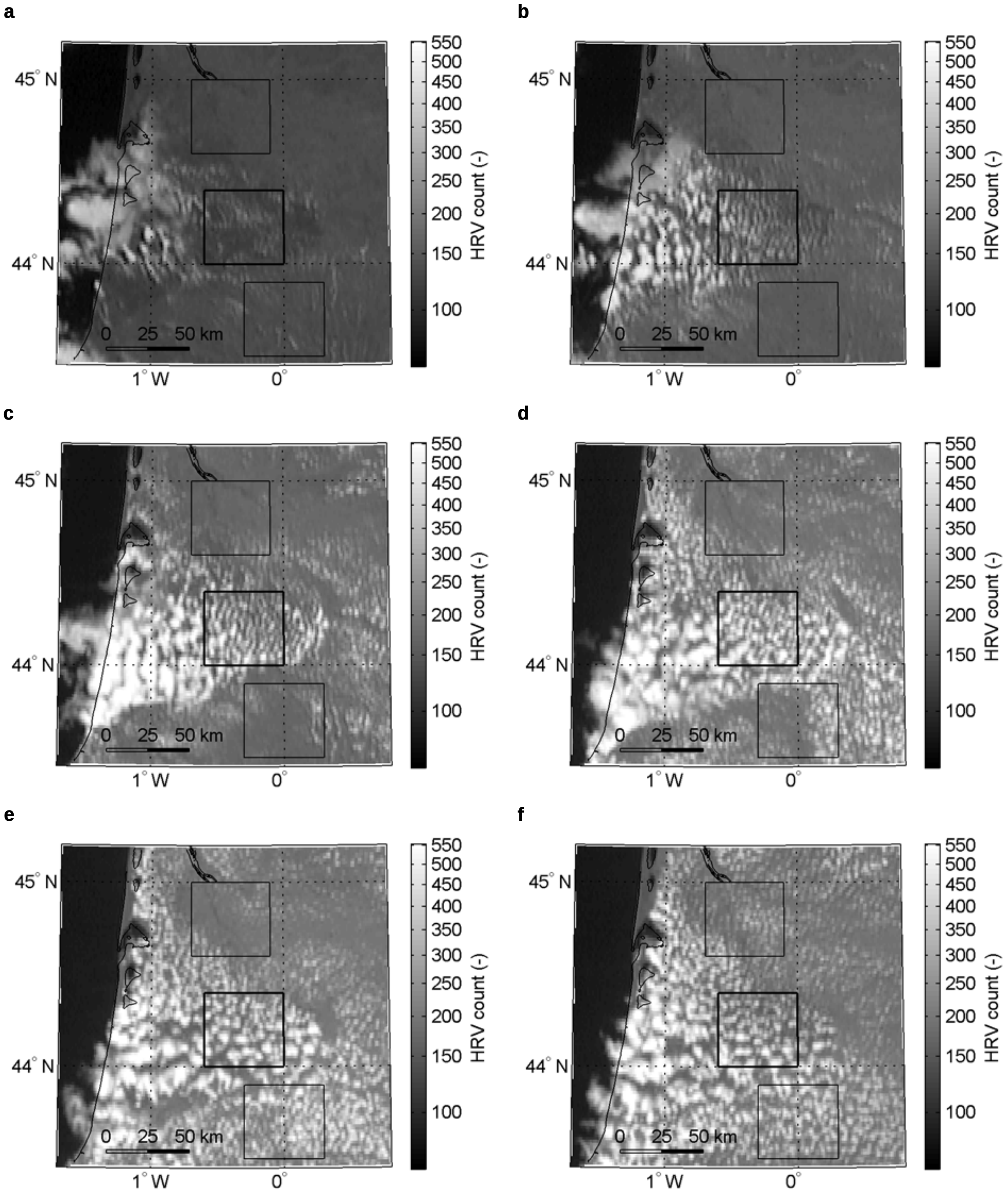

Supplementary Figure 7 | Snapshots of cloud cover development on 14 July 2008 over Landes. (**a**) 08 UTC, (**b**) 09 UTC, (**c**) 10 UTC, (**d**) 11 UTC, (**e**) 12 UTC, (**f**) 13 UTC. Images are based on Meteosat Second Generation high-resolution visible (HRV) imagery. Boxes indicate the location of forest (thick line) and non-forest (thin line) areas for visual reference.

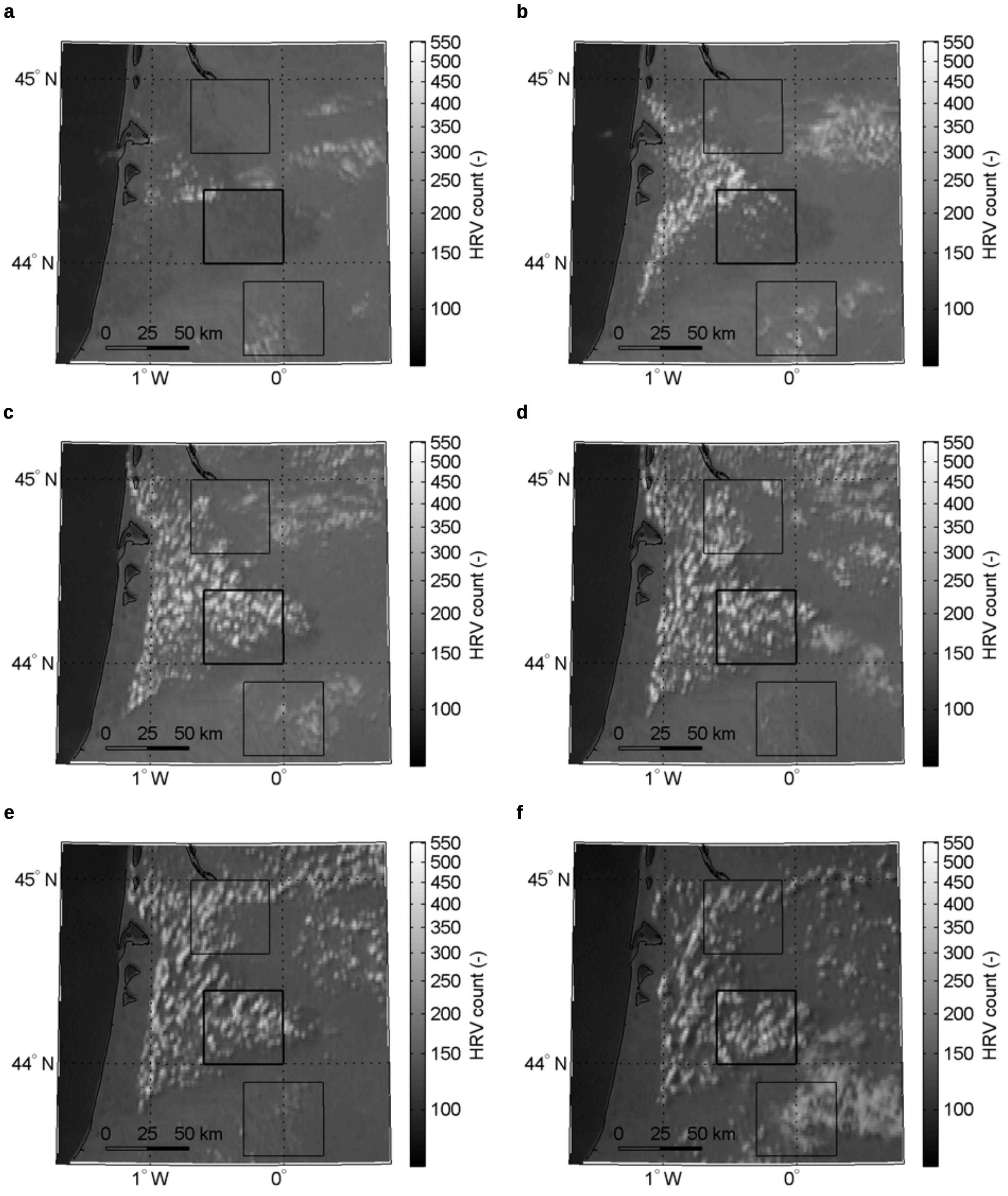

Supplementary Figure 8 | Snapshots of cloud cover development on 9 July 2013 over Landes. (**a**) 11 UTC, (**b**) 12 UTC, (**c**) 13 UTC, (**d**) 14 UTC, (**e**) 15 UTC, (**f**) 16 UTC. Images are based on Meteosat Second Generation high-resolution visible (HRV) imagery. Boxes indicate the location of forest (thick line) and non-forest (thin line) areas for visual reference.

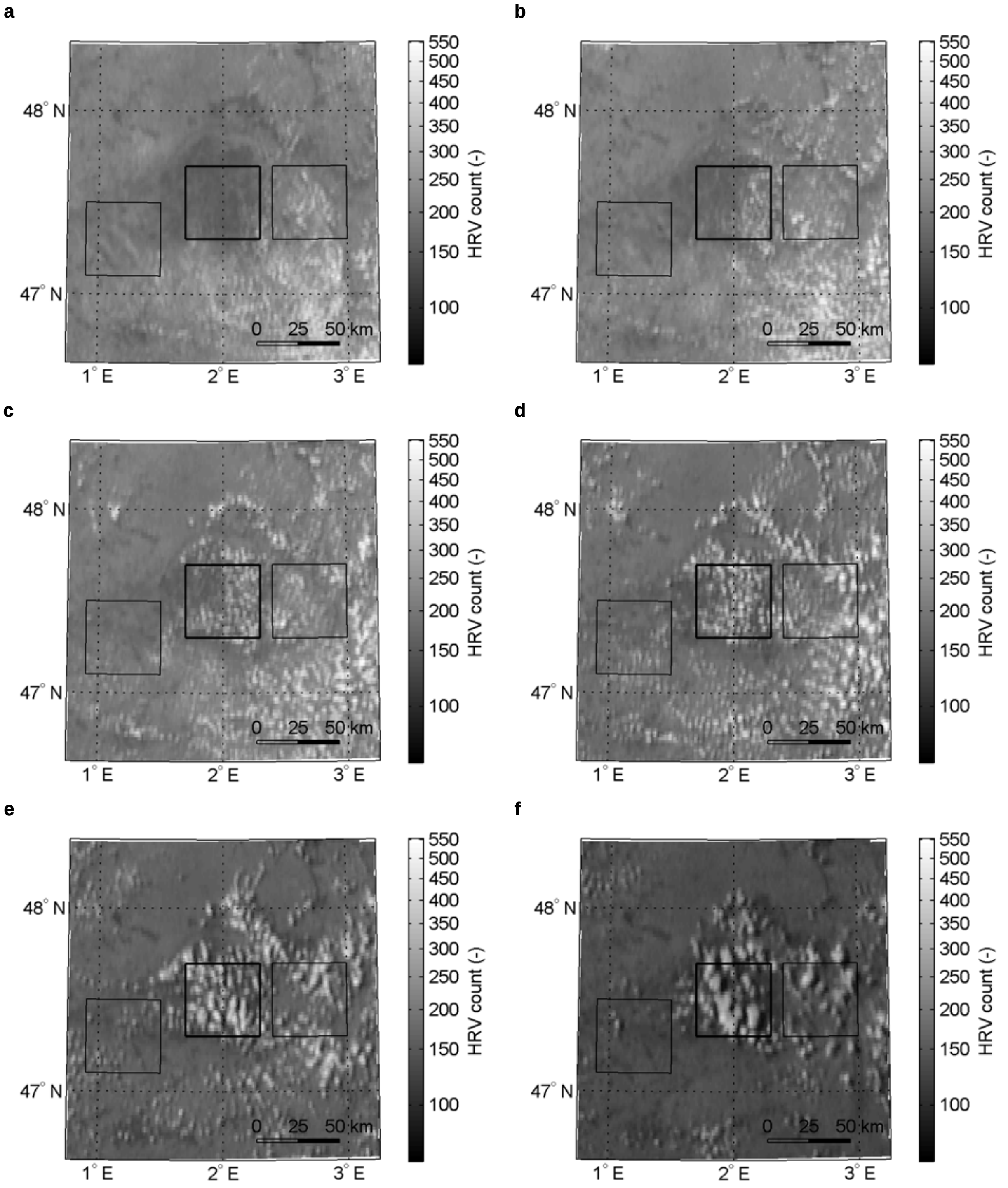

Supplementary Figure 9 | Snapshots of cloud cover development on 1 May 2012 over Sologne. (**a**) 11 UTC, (**b**) 12 UTC, (**c**) 13 UTC, (**d**) 14 UTC, (**e**) 15 UTC, (**f**) 16 UTC. Images are based on Meteosat Second Generation high-resolution visible (HRV) imagery. Boxes indicate the location of forest (thick line) and non-forest (thin line) areas for visual reference.

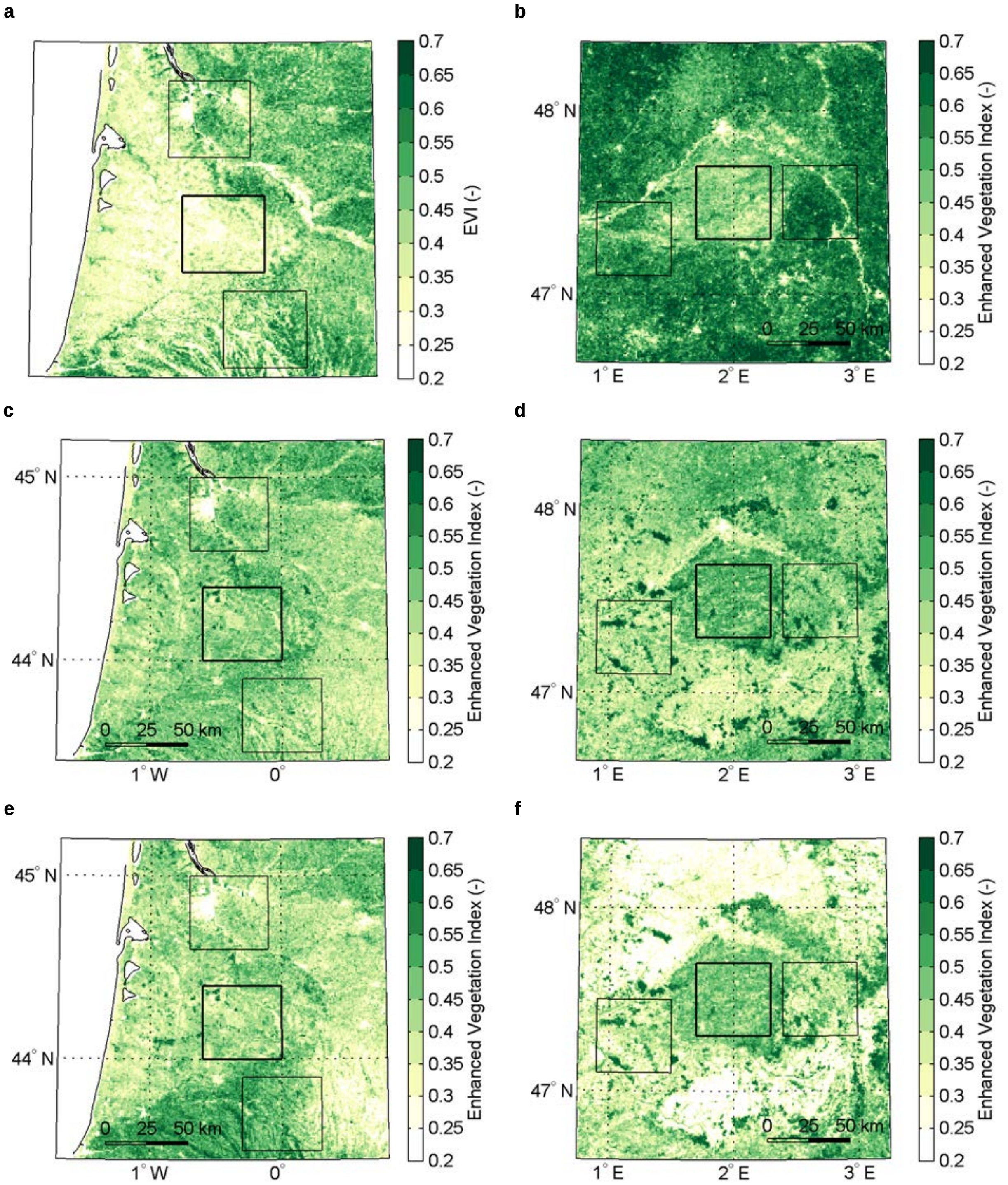

Supplementary Figure 10 | Seasonal development of vegetation activity. (**a,b**) Enhanced Vegetation Index (EVI) for day of year 121 for Landes (**a**) and Sologne (**b**). (**c,d**) EVI for day of year 169 for Landes (**c**) and Sologne (**d**). (**e,f**) EVI for day of year 169 for Landes (**e**) and Sologne (**f**). EVI is taken as averages over the years 2004–2013. Boxes indicate the location of forest (thick line) and non-forest (thin line) areas for visual reference.

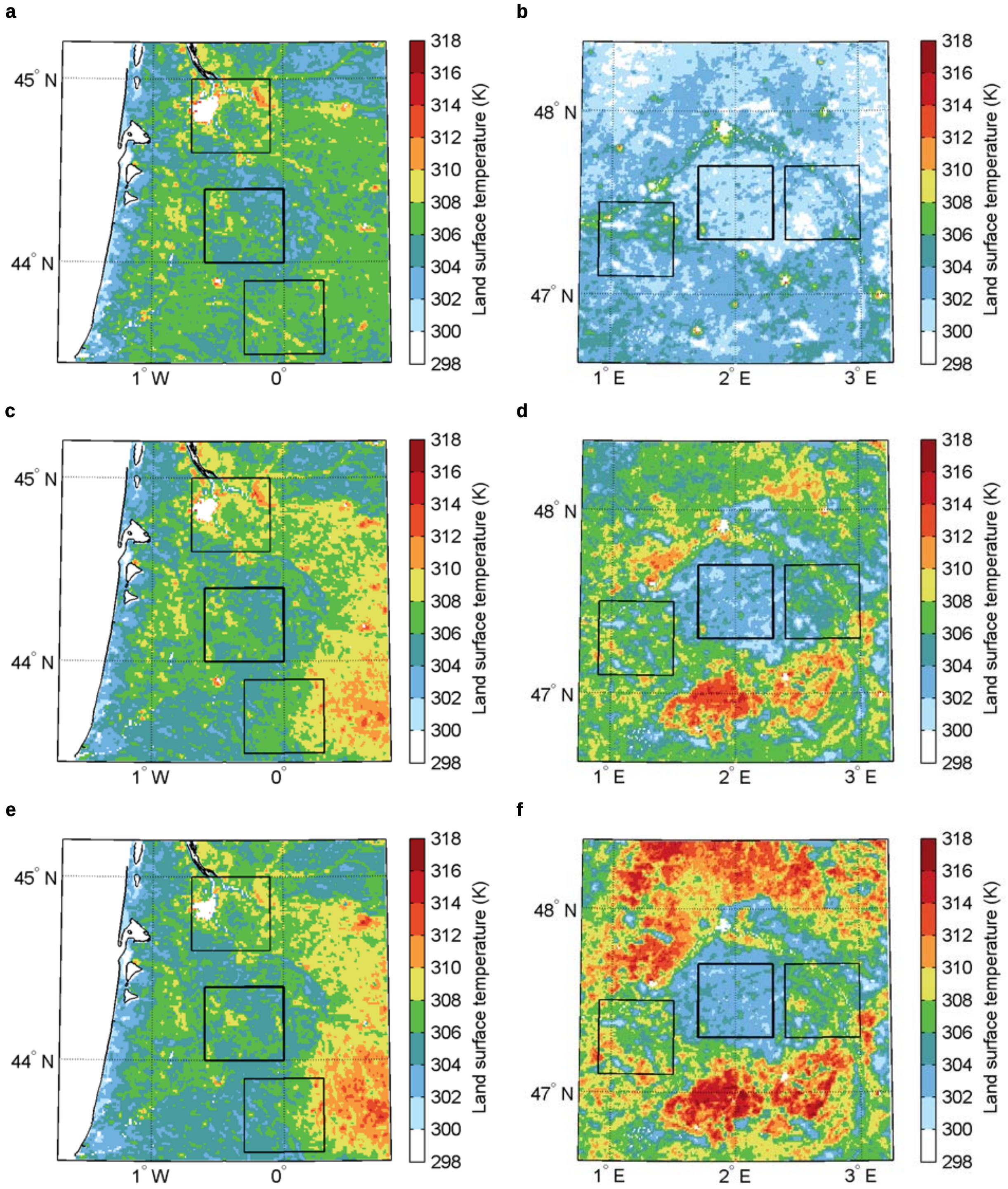

Supplementary Figure 11 | Seasonal development of land surface temperature. (**a,b**) Average June land surface temperature (LST) for Landes (**a**) and Sologne (**b**). (**c,d**) Average July LST for Landes (**c**) and Sologne (**d**). (**e,f**) Average August LST for Landes (**e**) and Sologne (**f**). LST is taken from MODIS Aqua and calculated as averages over the years 2002–2014 after screening for cloud presence and shading. Boxes indicate the location of forest (thick line) and non-forest (thin line) areas for visual reference.

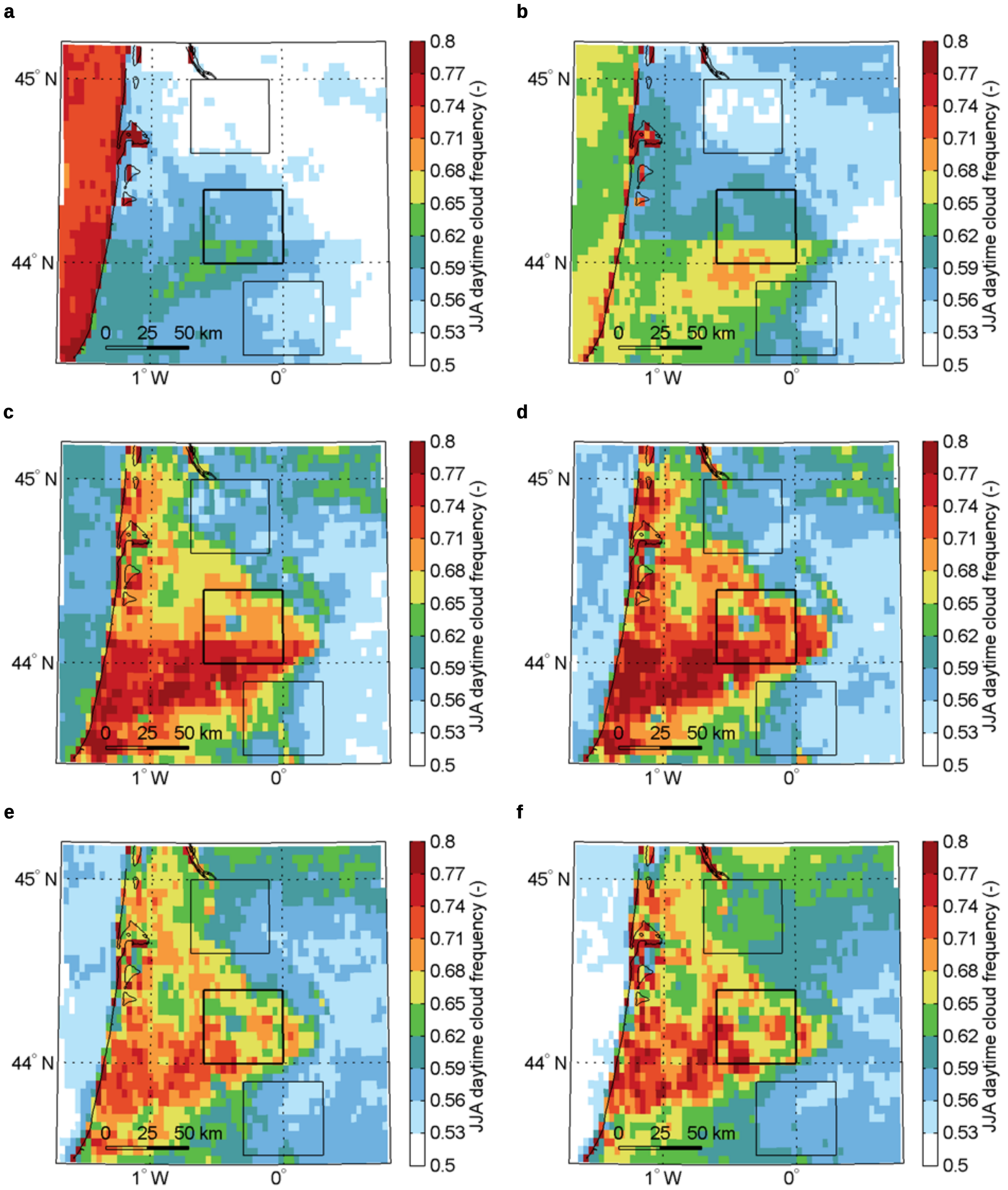

Supplementary Figure 12 | Diurnal evolution of average summer cloud frequency for Landes. (**a**) 06 UTC, (**b**) 07 UTC, (**c**) 08 UTC, (**d**) 09 UTC, (**e**) 10 UTC, (**f**) 11 UTC. Cloud frequency is based on the Cloud Physical Properties algorithm and taken as average over June–August for the years 2004–2008. Boxes indicate the location of forest (thick line) and non-forest (thin line) areas for visual reference. Note that the first peak in the forest-non forest contrast occurs around 09 UTC.

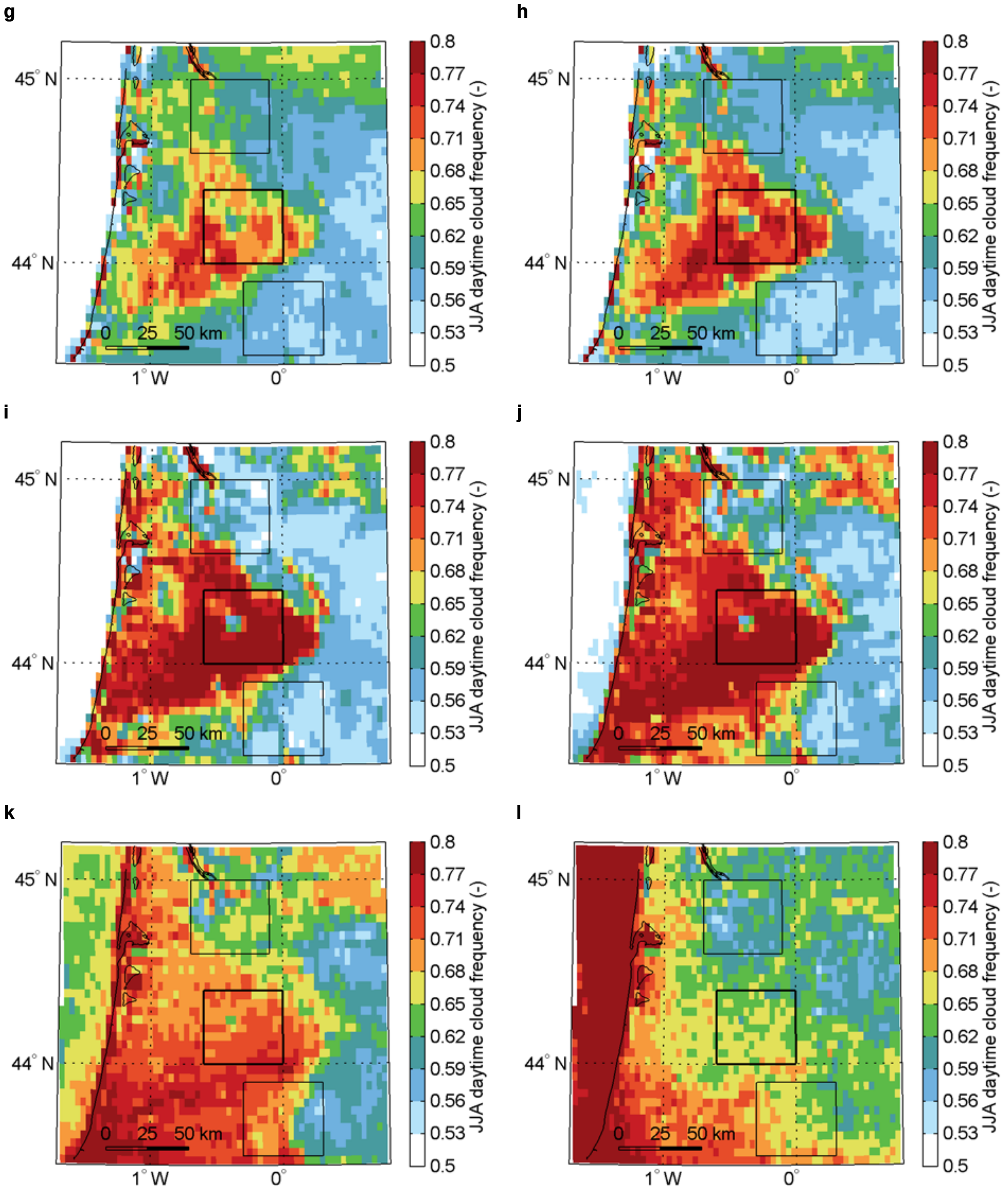

Supplementary Figure 12 (cont.) | Diurnal evolution of average summer cloud frequency for Landes. (**g**) 13 UTC, (**h**) 14 UTC, (**i**) 15 UTC, (**j**) 16 UTC, (**k**) 17 UTC, (**l**) 18 UTC. Cloud frequency is based on the Cloud Physical Properties algorithm and taken as average over June-August for the years 2004–2008. Boxes indicate the location of forest (thick line) and non-forest (thin line) areas for visual reference.

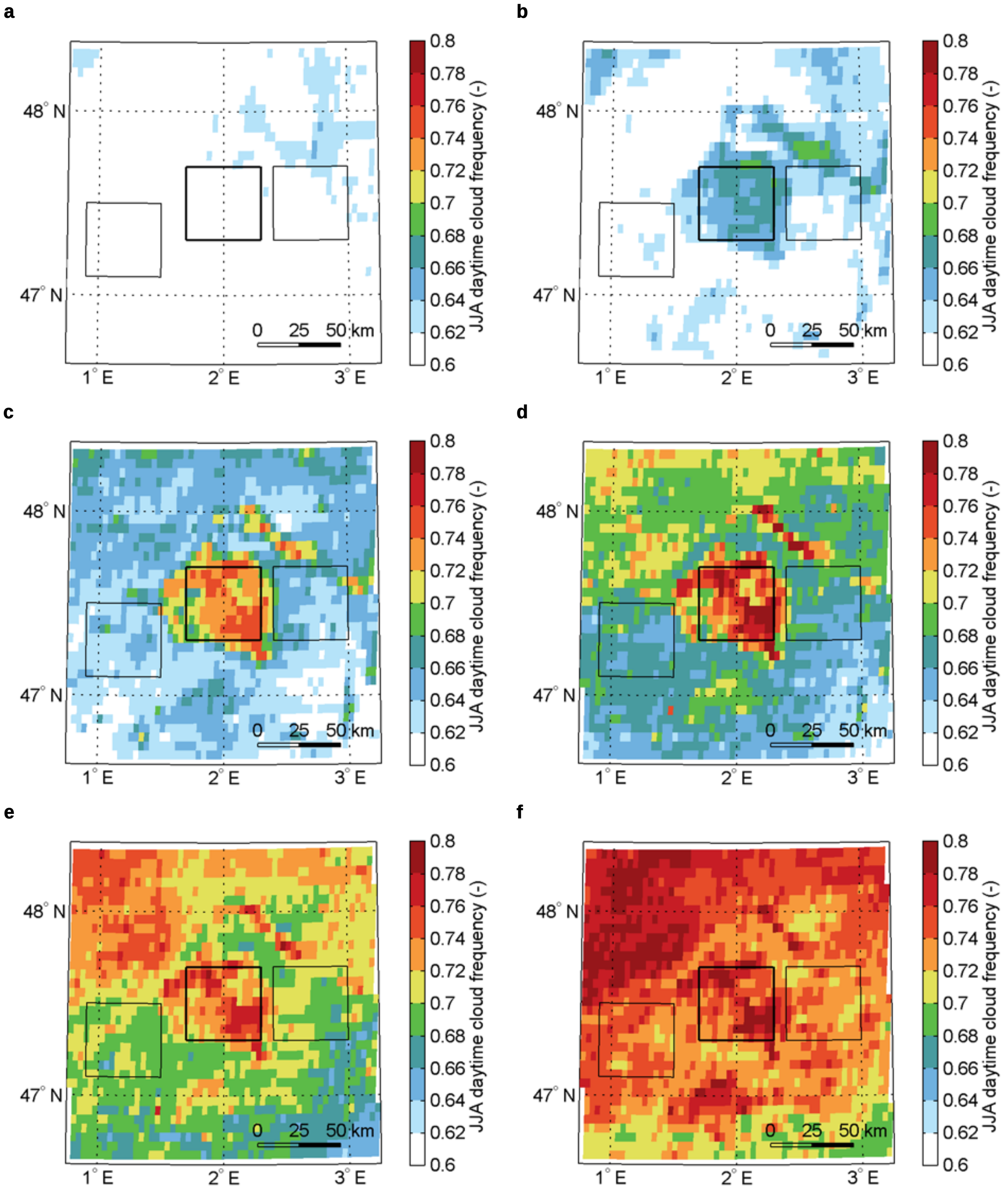

Supplementary Figure 13 | Diurnal evolution of average summer cloud frequency for Sologne. (**a**) 06 UTC, (**b**) 07 UTC, (**c**) 08 UTC, (**d**) 09 UTC, (**e**) 10 UTC, (**f**) 11 UTC. Cloud frequency is based on the Cloud Physical Properties algorithm and taken as average over June-August for the years 2004–2008. Boxes indicate the location of forest (thick line) and non-forest (thin line) areas for visual reference. Note that the first peak in the forest-non forest contrast occurs around 09 UTC.

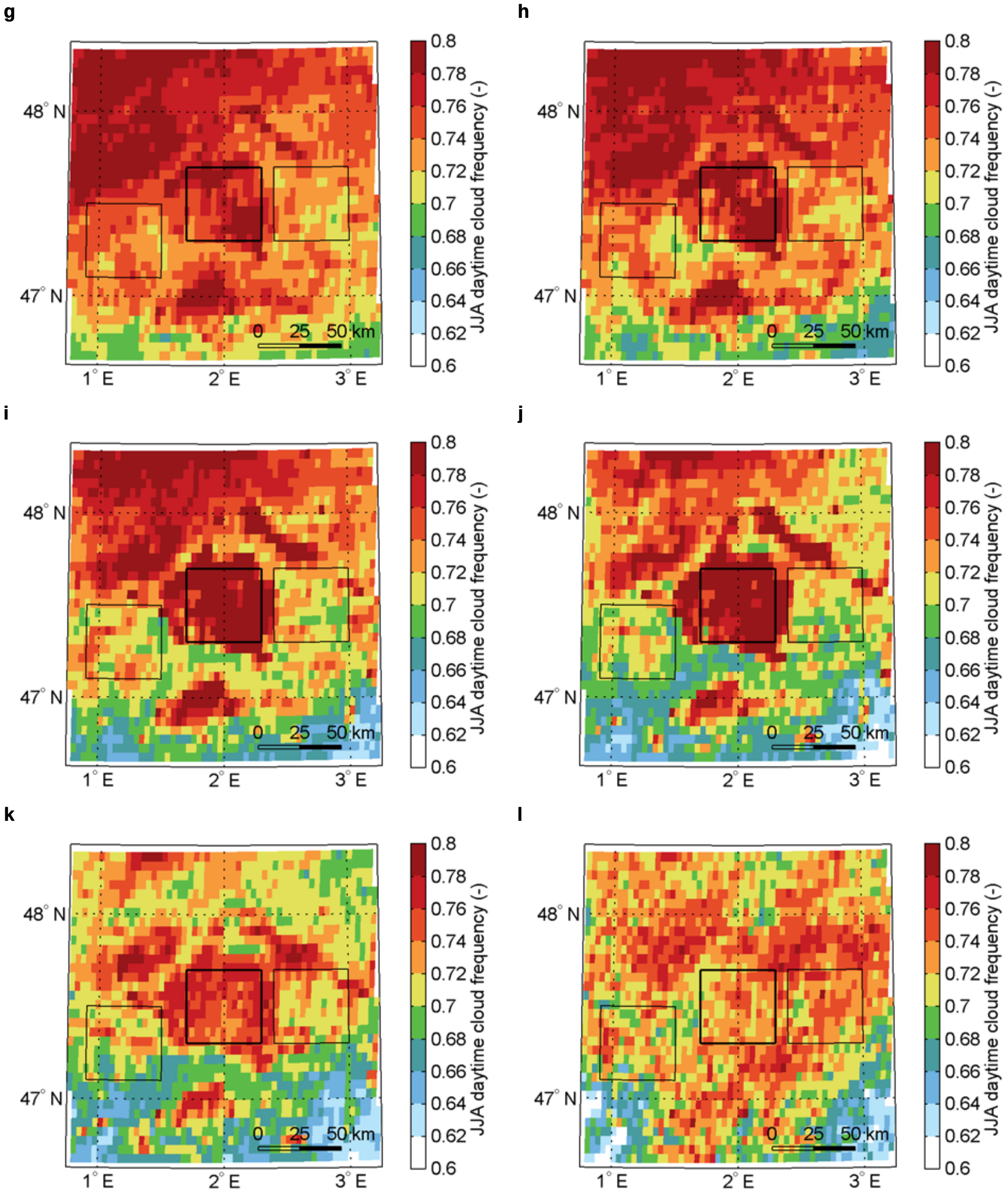

Supplementary Figure 13 (cont.) | Diurnal evolution of average summer cloud frequency for Sologne. (**g**) 13 UTC, (**h**) 14 UTC, (**i**) 15 UTC, (**j**) 16 UTC, (**k**) 17 UTC, (**l**) 18 UTC. Cloud frequency is based on the Cloud Physical Properties algorithm and taken as average over June-August for the years 2004–2008. Boxes indicate the location of forest (thick line) and non-forest (thin line) areas for visual reference.

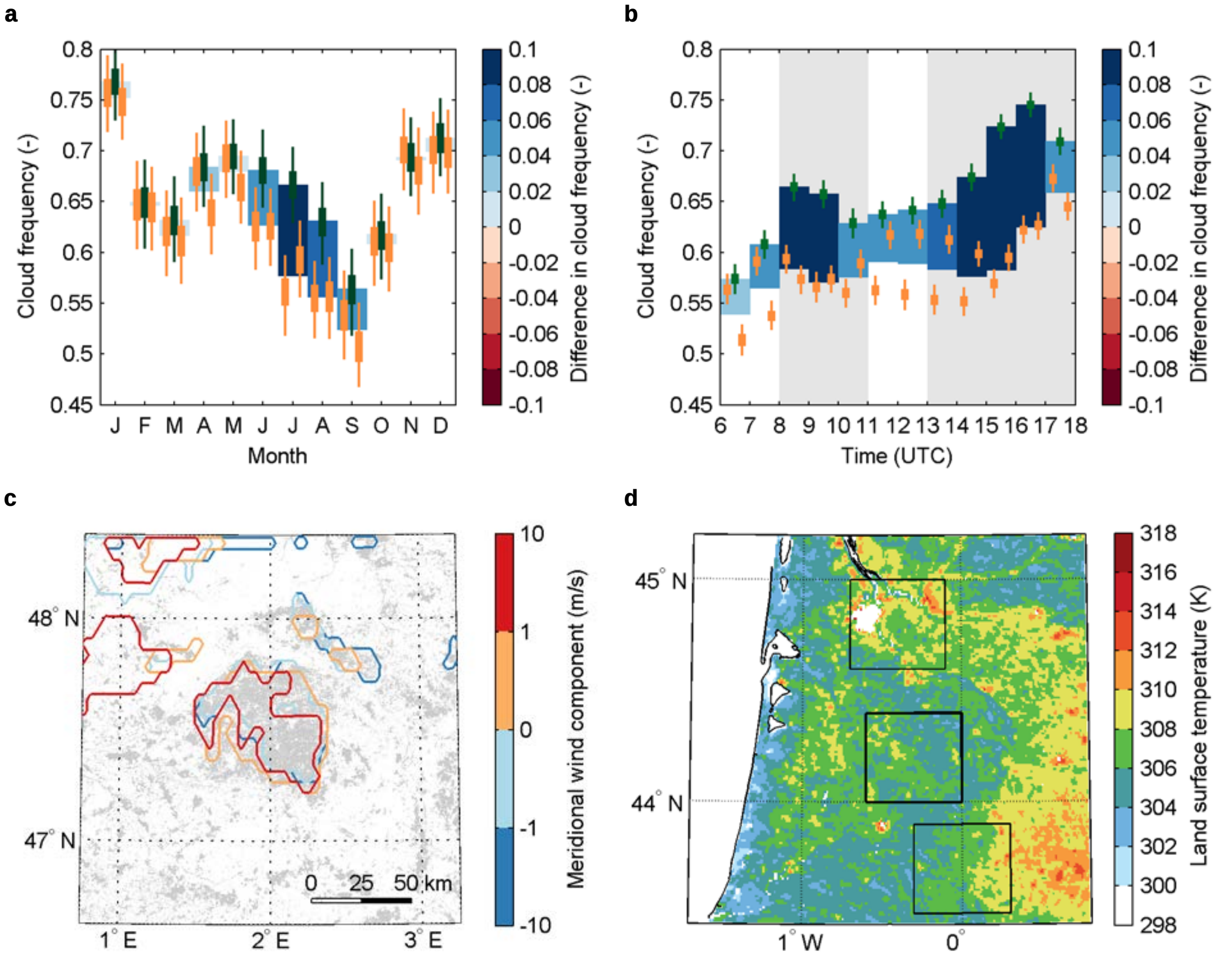

Supplementary Figure 14 | Temporal and spatial patterns of preferred cloud occurrence over forest. (**a,b**) Seasonal (**a**) and diurnal (**b**) evolution of mean June-August (JJA) difference in cloud frequency for Landes region. (**c**) Impact of regional (10 m) meridional wind component on JJA cloud frequency for Sologne. (**d**) Average July land surface temperature (2002–2014) from MODIS Aqua for Landes. In **a** and **b**, thick (thin) vertical lines indicate 50 (95) percentile intervals obtained from bootstrapping for the forest (green) and non-forest boxes (orange). Shading indicates periods of significant difference at the 95% confidence level. In **c**, contours represent 90th percentiles to account for differences in mean cloud frequency with wind conditions. In **d**, boxes indicate the location of forest (thick line) and non-forest (thin line) areas for visual reference. Note that positive meridional wind is wind blowing from south to north. All plots are based on the Cloud Physical Properties algorithm.

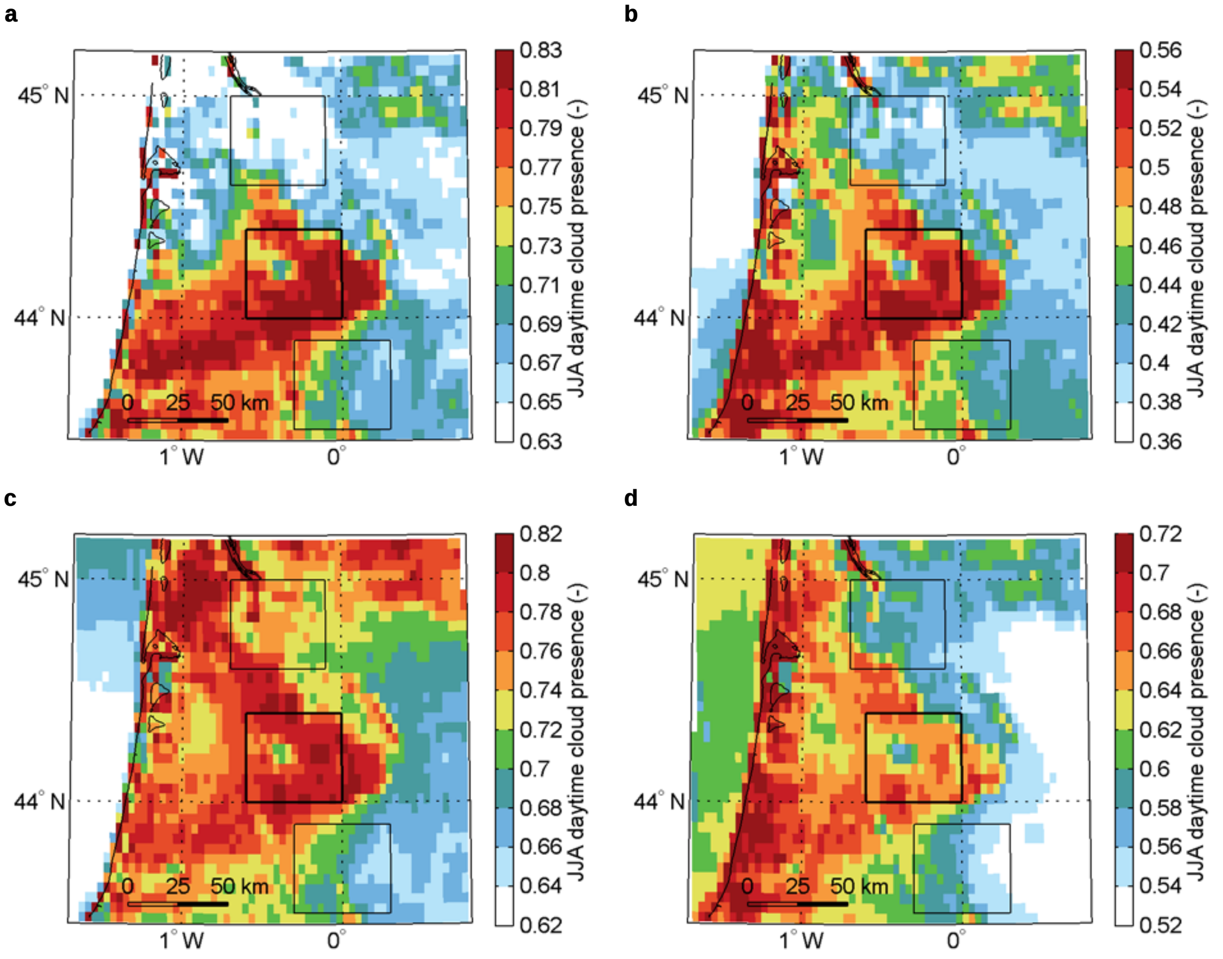

Supplementary Figure 15 | Impact of wind direction on summer cloud frequency for Landes. (**a**) Cloud frequency under North-Westerly winds. (**b**) Cloud frequency under North-Easterly winds. (**c**) Cloud frequency under South-Westerly winds. (**d**) Cloud frequency for South-Easterly winds. Cloud frequency is based on the Cloud Physical Properties algorithm and taken as average over June-August for the years 2004–2013. Boxes indicate the location of forest (thick line) and non-forest (thin line) areas for visual reference.

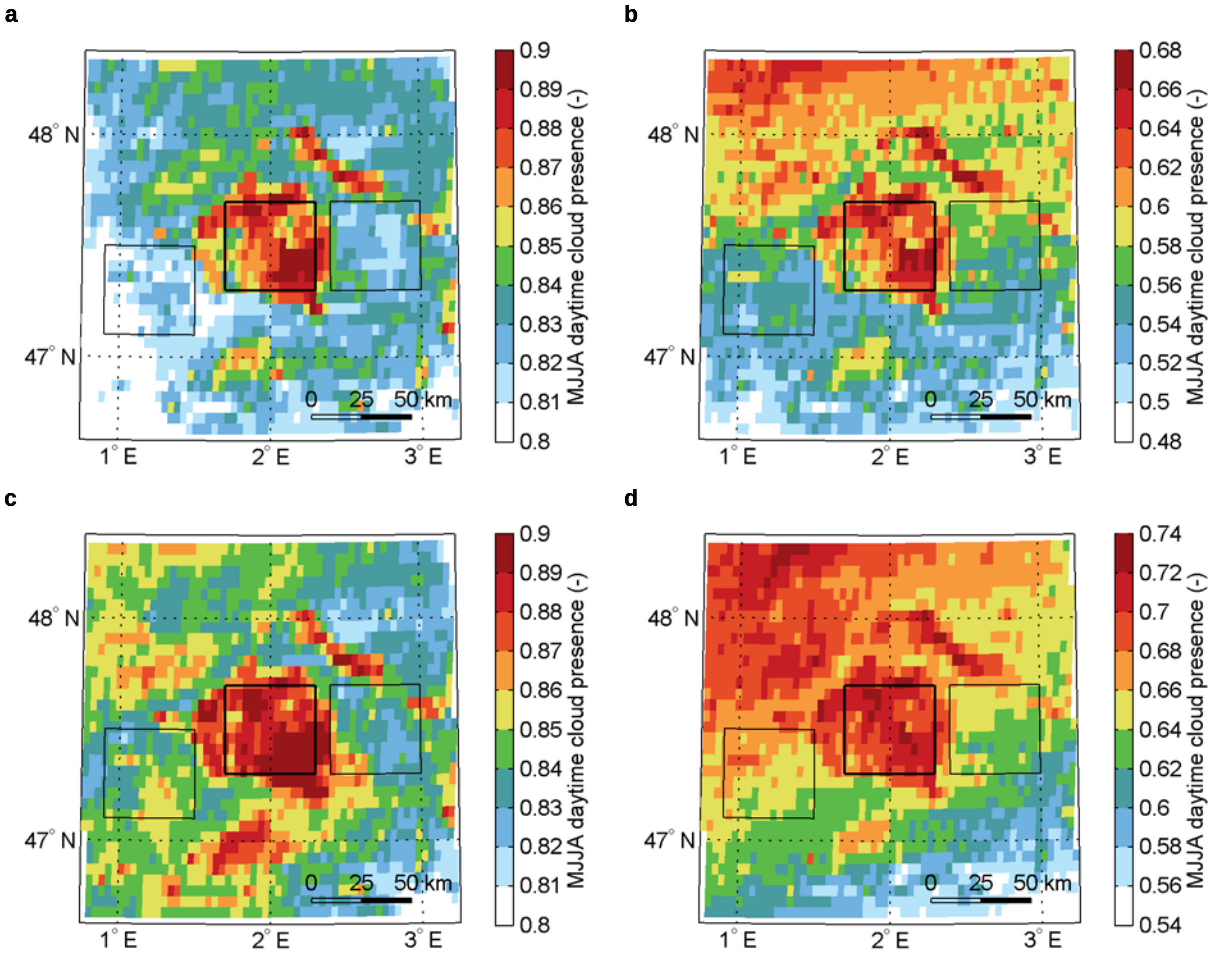

Supplementary Figure 16 | Impact of wind direction on summer cloud frequency for Sologne. (**a**) Cloud frequency under North-Westerly winds. (**b**) Cloud frequency under North-Easterly winds. (**c**) Cloud frequency under South-Westerly winds. (**d**) Cloud frequency for South-Easterly winds. Cloud frequency is based on the Cloud Physical Properties algorithm and taken as average over June-August for the years 2004–2013. Boxes indicate the location of forest (thick line) and non-forest (thin line) areas for visual reference.

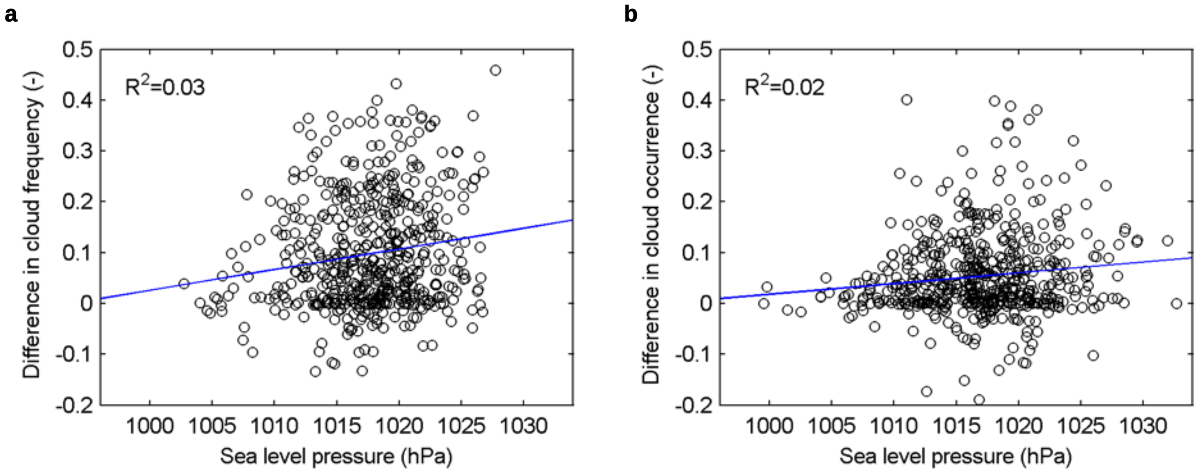

Supplementary Figure 17 | Impact of sea level pressure on mean difference in daily average summer cloud frequency. (**a**) Landes, (**b**) Sologne. Blue line indicates linear fit. Cloud frequency is based on the Cloud Physical Properties algorithm and taken over June–August for the years 2004–2008. Non-forest conditions were taken as the average over the two boxes. Sea level pressure data were obtained from ERA-Interim.

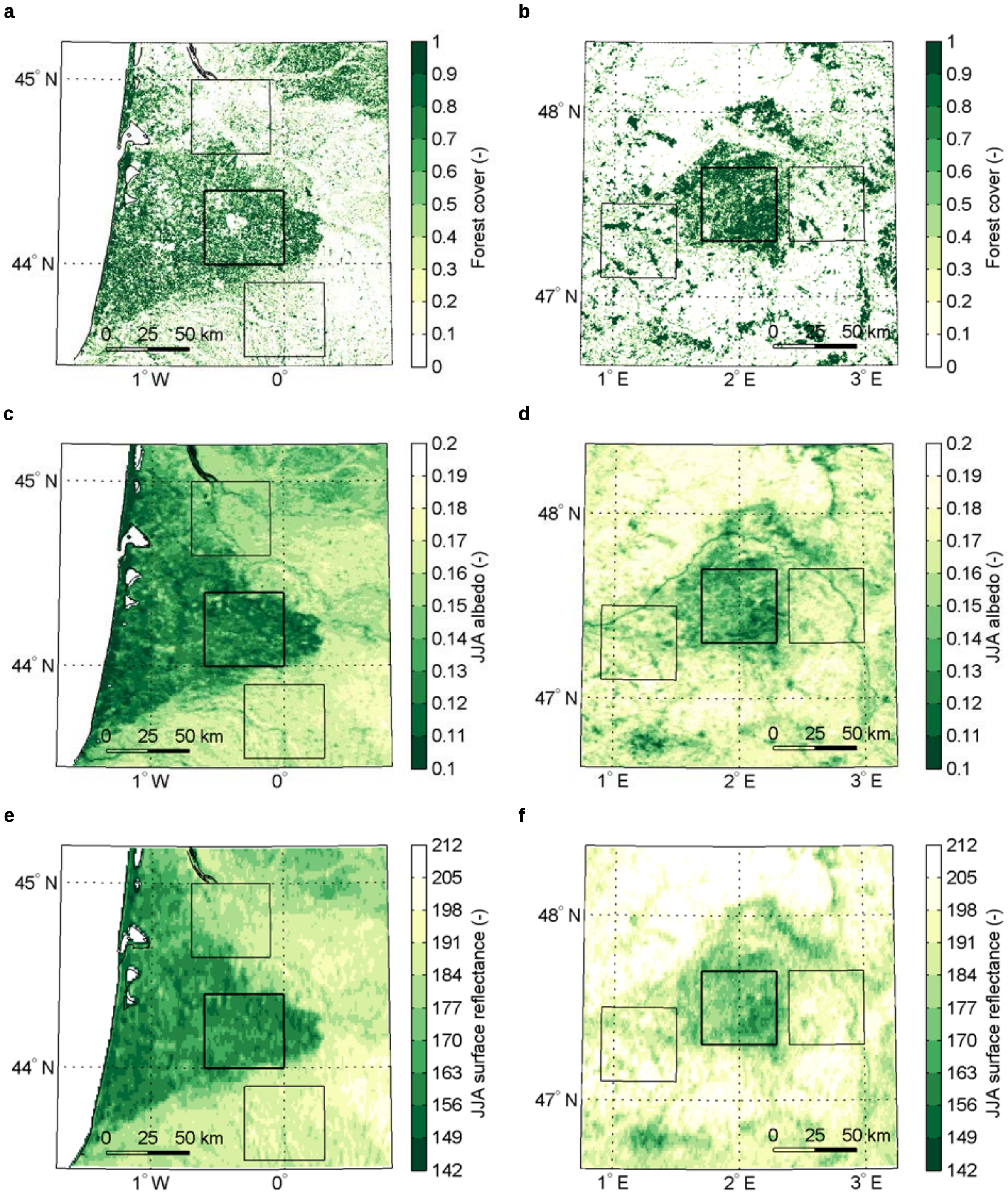

Supplementary Figure 18| Forest cover and land surface albedo. (**a,b**) Forest cover (data from JRC) for Landes (**a**) and Sologne (**b**). (**c,d**) June–August average black-sky shortwave albedo from MODIS for Landes (**c**) and Sologne (**d**). (**e,f**) High Resolution Visible (MSG-HRV) clear-sky radiation resulting from the empirical cumulative distribution function analysis on Meteosat Second Generation channel 12 data (see Methods) for Landes (**e**) and Sologne (**f**). Boxes indicate the location of forest (thick line) and non-forest (thin line) areas for visual reference. Note the close resemblance between the distribution of MODIS albedo and the clear-sky radiation in spite of the fact that the latter has been inferred on a pixel-by-pixel basis.
